# Supplementary material for: Asynchronous magnetic resonance elastography: Shear wave speed reconstruction using noise correlation of incoherent waves
Source: Magn Reson Med. 2022 Oct 27;89(3):990–1001. doi: 10.1002/mrm.29502 (PMC9792433; doi:10.1002/mrm.29502)
Supplement: Supplementary file 1 — DATA S1 MATLAB code used to generate the numerical simulations of Figure 1 [file MRM-89-990-s002.zip › k-Wave/helpfiles/example_sd_directional_array_elements.html]

Focussed 2D Array With Directional Elements Example (k-Wave)


# Focussed 2D Array With Directional Elements Example

This example demonstrates the use of k-Wave to compute the outputs from a curved detector array which consists of several elements, each of which consists of a number of grid points.

- Open the file in the MATLAB Editor
- Run the file in MATLAB

## Contents

- Define a focussed array of directional elements
- Using an infinitely wide line source
- Running the simulation
- Plotting

## Define a focussed array of directional elements

To start with, a curved detector array is defined using the `makeCircle` function.

```
% define a semicircular sensor centred on the grid
semicircle_radius = 65; % [grid points]
arc = makeCircle(Nx, Ny, Nx/2, Ny/2, semicircle_radius, pi);
```

The grid points making up this curved array are divided up between a number of elements. This is achieved by calculating the angles from each grid point to the focus of the array and grouping the grid points with similar angles.

```
% find total number and indices of the grid points constituting the
% semicircle
arc_indices = find(arc == 1);
Nv = length(arc_indices);

% calculate angles between grid points in the arc and the centre of the
% grid
arc_angles = atan((kgrid.y(arc_indices)) ./ kgrid.x(arc_indices));

% sort the angles into ascending order, and adjust the indices accordingly
[sorted_arc_angles, sorted_index] = sort(arc_angles);
sorted_arc_indices = arc_indices(sorted_index);

% divide the semicircle into Ne separate sensor elements
Ne = 13;
sensor.mask = zeros(Nx, Ny);
for loop = 1:Ne
    
    % get the indices of the grid points belonging to the current element
    % (there is a two grid point gap between the elements)
    voxel_indices = sorted_arc_indices(floor((loop - 1) * Nv / Ne) + ...
        2:floor(loop * Nv / Ne) - 1);
    
    % add the element to the sensor.mask
    sensor.mask(voxel_indices) = 1;
    
end
```


## Using an infinitely wide line source

To model a plane wave source parallel to one of the grid dimensions, two steps are necessary. First, `source.p_mask` is set so that it stretches across the domain.

```
source.p_mask = zeros(Nx, Ny);
source.p_mask(140, :) = 1;
```

Secondly, the inputs to the simulation are set so that the perfectly matched layer is turned off on the two sides that `source.p_mask` meets.

```
input_args = {'PMLAlpha', [2, 0], 'DisplayMask', display_mask, 'PlotScale', [-0.75, 0.75]};
```

## Running the simulation

The simulation is run and the time series are measured for every non-zero grid point in `sensor.mask`. These time series are then allocated to their relevant elements, and averaged together.

```
sensor_data = kspaceFirstOrder2D(kgrid, medium, source, sensor, input_args{:});

% split up the recorded data between the elements
element_data = zeros(Ne, kgrid.Nt);
for loop = 1:Ne
    
    % find indices of the sensor grid points in the sensor mask
    sensor_indices = find(sensor.mask == 1);
    
    % extract indices of the grid points belonging to the current element
    voxel_indices = sorted_arc_indices(floor((loop - 1) * Nv / Ne) + ...
        2:floor(loop * Nv / Ne) - 1);
    
    % indices of sensor_data that refer to the data for this element
    data_indices = zeros(length(voxel_indices), 1);
    for loop2 = 1:length(voxel_indices)
        data_indices(loop2) = find(sensor_indices == voxel_indices(loop2));
    end    

    % for one element per loop, average the time series from each of the
    % element's grid points to give one time series for the whole element
    element_data(loop, :) = mean(sensor_data(data_indices, :), 1);
    
end
```

The time series corresponding to the different elements are plotted. The directionality introduced by the large size of the detector (because it averages the incoming wave over its area) is clearly seen.
